# Inferior Pole Sleeve Fracture of the Patella in an Adolescent: A Case Report

**DOI:** 10.7759/cureus.33494

**Published:** 2023-01-07

**Authors:** Koshiro Shimasaki, Masafumi Uesugi, Takahiro Kobayashi, Haruka Tanaka, Harumitsu Ichimura

**Affiliations:** 1 Department of Orthopaedic Surgery, Ibaraki Seinan Medical Center Hospital, Sakai, JPN

**Keywords:** pull-out, pediatric sleeve fracture, patella fracture, avulsion fracture, pediatrics

## Abstract

An 11-year-old boy was admitted to our hospital due to severe pain in his right knee when he landed after jumping over a vaulting box. A plain X-ray image and computed tomography scan showed an avulsion fracture of the lower pole of the patella and patella alta. Furthermore, magnetic resonance imaging (MRI) revealed an articular cartilage lesion and rupture between the inferior pole of the patella and the patella tendon. We diagnosed a sleeve fracture of the patella and performed surgical treatment. Open reduction and internal fixation were performed by the pull-out technique using transosseous no. 2 MaxBraid™​​​​​​ ​(Zimmer Biomet, Tokyo, Japan) sutures. While postoperative weight-bearing was permitted, the knee joint was immobilized in a brace for four weeks. Three months of postoperative assessment revealed excellent functional outcomes.

## Introduction

The first case of rupture of the knee’s extensor mechanism, which involves a patella avulsion fracture with a lesion of the articular cartilage, periosteum, and patellar retinaculum, was reported in 1979 and termed a patella sleeve fracture [1]. In general, a patella sleeve fracture is rare, and it has very few characteristic radiographic features, thereby increasing the risk of a delayed or missed diagnosis in an emergency room, which may be disadvantageous for the patient. Moreover, it is often difficult to treat this type of trauma because the distal bony fragments are small. This paper highlights the necessity of understanding the distinctive anatomy of an immature patella during childhood, the importance of an early and accurate diagnosis, and obtaining anatomic repositioning and stable fixation.

Herein, we present the case of an inferior pole sleeve fracture of the patella in an adolescent and describe its successful outcome with open reduction and internal fixation using the pull-out technique with no. 2 MaxBraid™ (Zimmer Biomet, Tokyo, Japan) sutures.

## Case presentation

An 11-year-old boy was brought to our hospital as he was suffering from severe pain in his right knee after he landed on the ground after vaulting over a box during a physical education class. There was nothing significant about his medical, family, exercise, and psychosocial histories. Swelling and a subcutaneous dimple at the distal aspect of his patella were observed. He could not independently extend his knee fully, with an extension lag of approximately 30 degrees, owing to severe pain.

**Figure 1:**
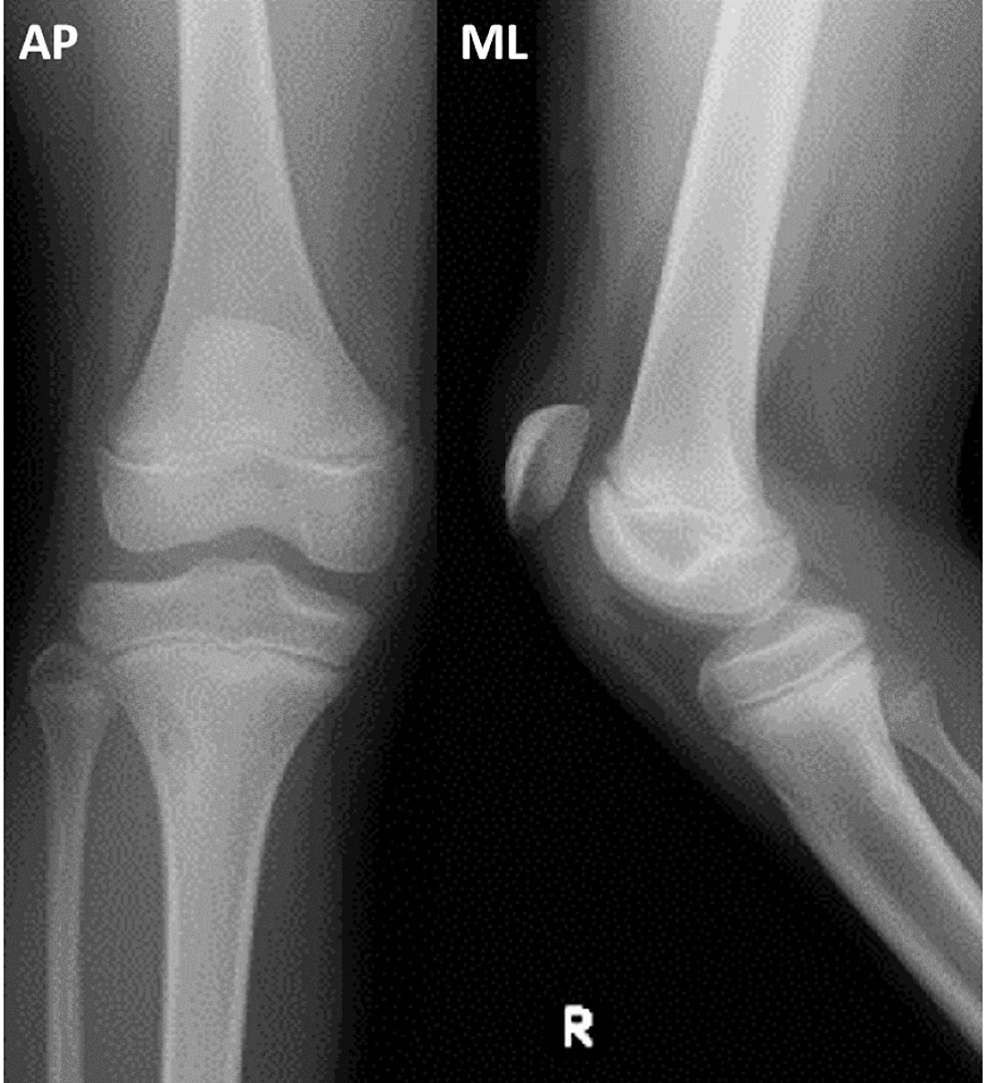
Plain radiographs on the first visit. Plain radiographs at the first visit revealed a high-riding patella without any obvious bone fracture around the knee.

**Figure 2:**
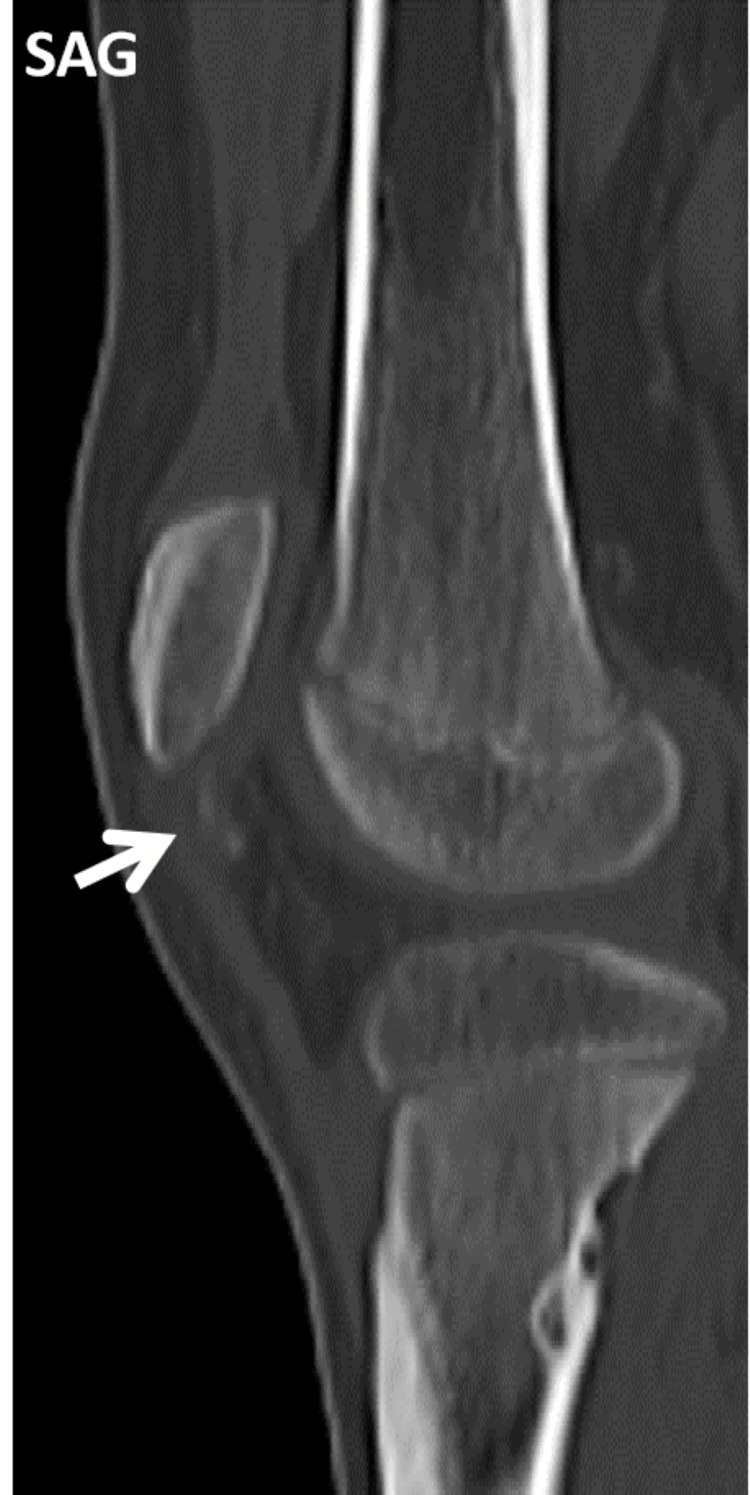
Computed tomography image at the first visit. Computed tomography image in a sagittal plane at the first visit revealed an apparent fragment of the lower pole of the patella (white arrow).

Radiographs taken at the emergency department revealed a high-riding patella accompanied by a small and thin fragment of the lower pole of the patella found on computed tomography (CT) (Figures 1-2).

**Figure 3:**
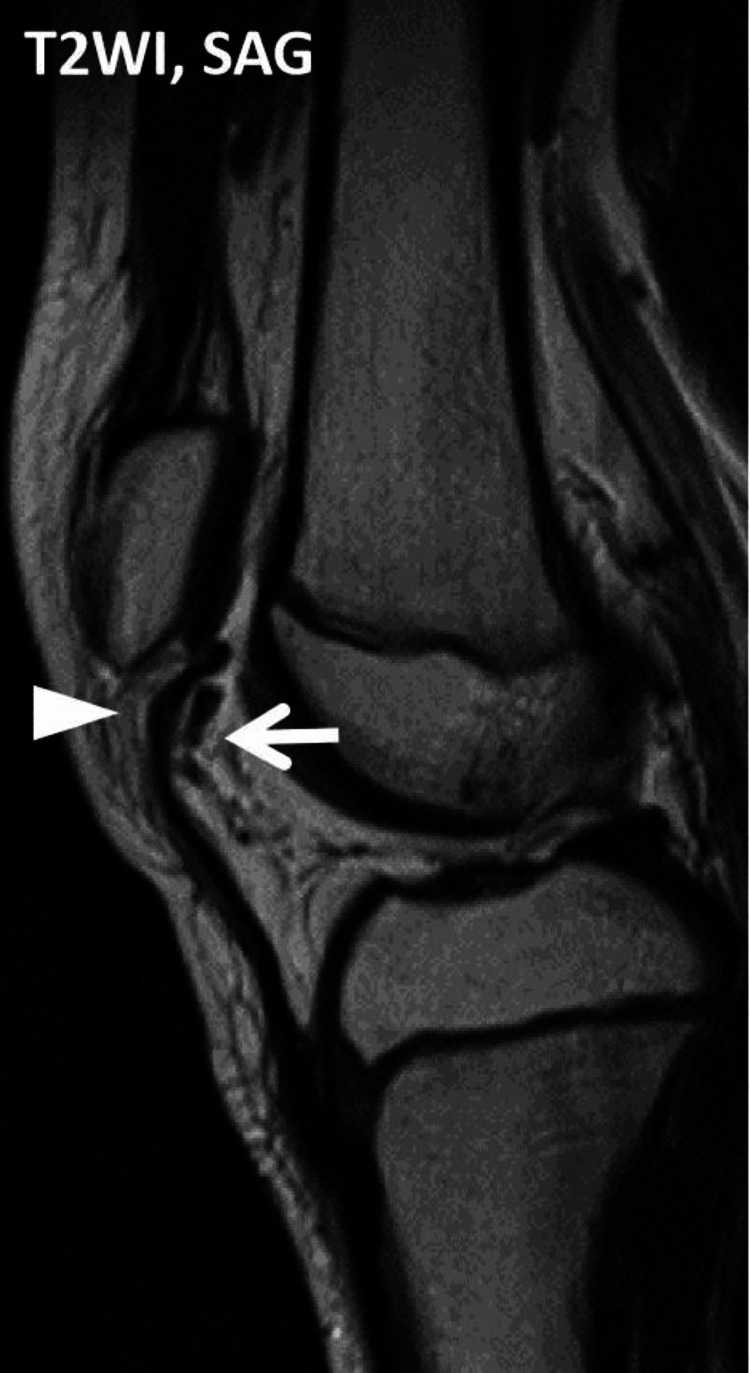
Magnetic resonance imaging at the first visit. T2-weighted magnetic resonance imaging in a sagittal plane at the first visit revealed complete disruption of the patellar ligament (white arrowhead) and an osteochondral fragment avulsed from the patella (white arrow).

Magnetic resonance imaging (MRI) revealed complete disruption of the patellar ligament with an osteochondral fragment avulsed from the patella and attached to the ligament without any other abnormality in the intra-articular area (Figure 3). There was no abnormality around the intra-articular area.

**Figure 4:**
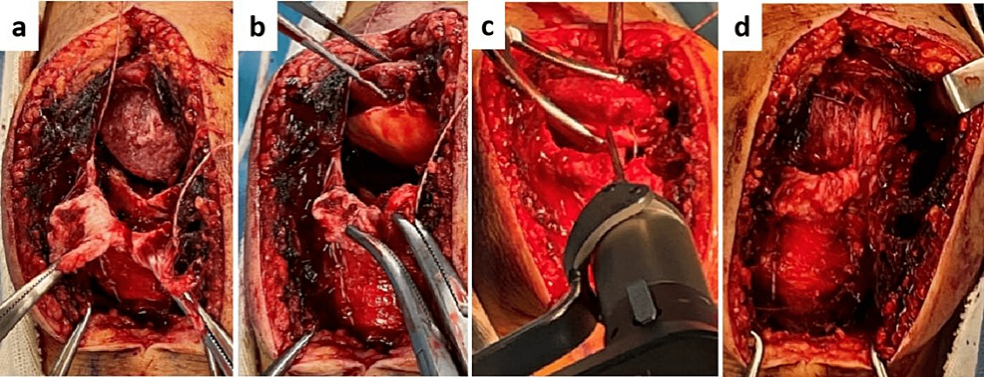
Surgical findings. Complete disruption of the patella ligament from the inferior pole of the patella and an avulsion fracture of the inferior pole (a) and articular cartilage of the patella (b), indicating a rupture of the extensor mechanism of the knee. The pull-out suturing technique was performed (c, d).

Considering these clinical findings, we diagnosed a sleeve fracture of the patella and proceeded with surgical treatment. Perioperatively, complete disruption of the patella ligament from the inferior pole of the patella and an avulsion fracture of the inferior pole (3 mm) and articular cartilage of the patella (8 mm) (a rupture of the extensor mechanism of the knee) were noted (Figure 4).

**Figure 5:**
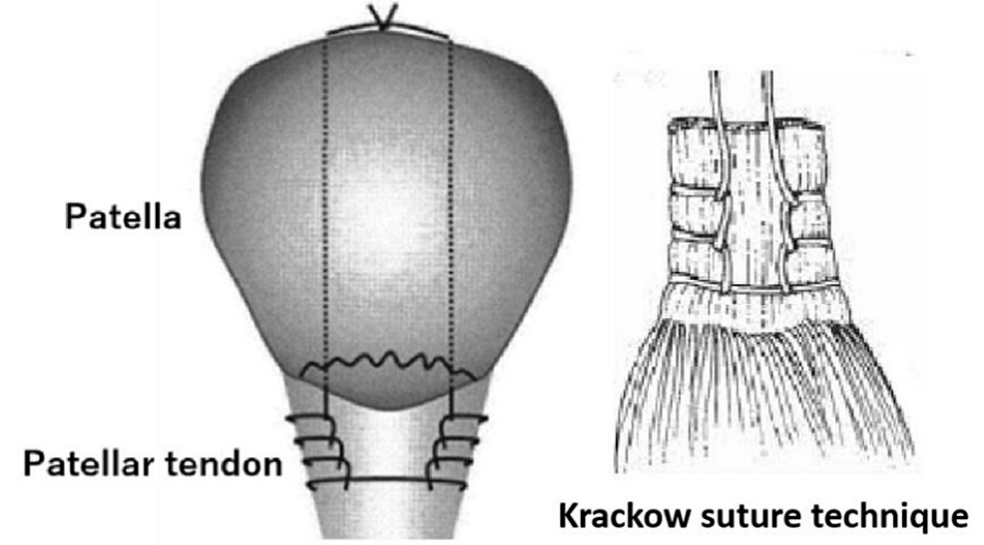
Operative procedure. The pull-out technique was used with transosseous no. 2 MaxBraid™ sutures. Krackow sutures were also performed with a continuous locking loop technique commonly used in tendon repair.

**Figure 6:**
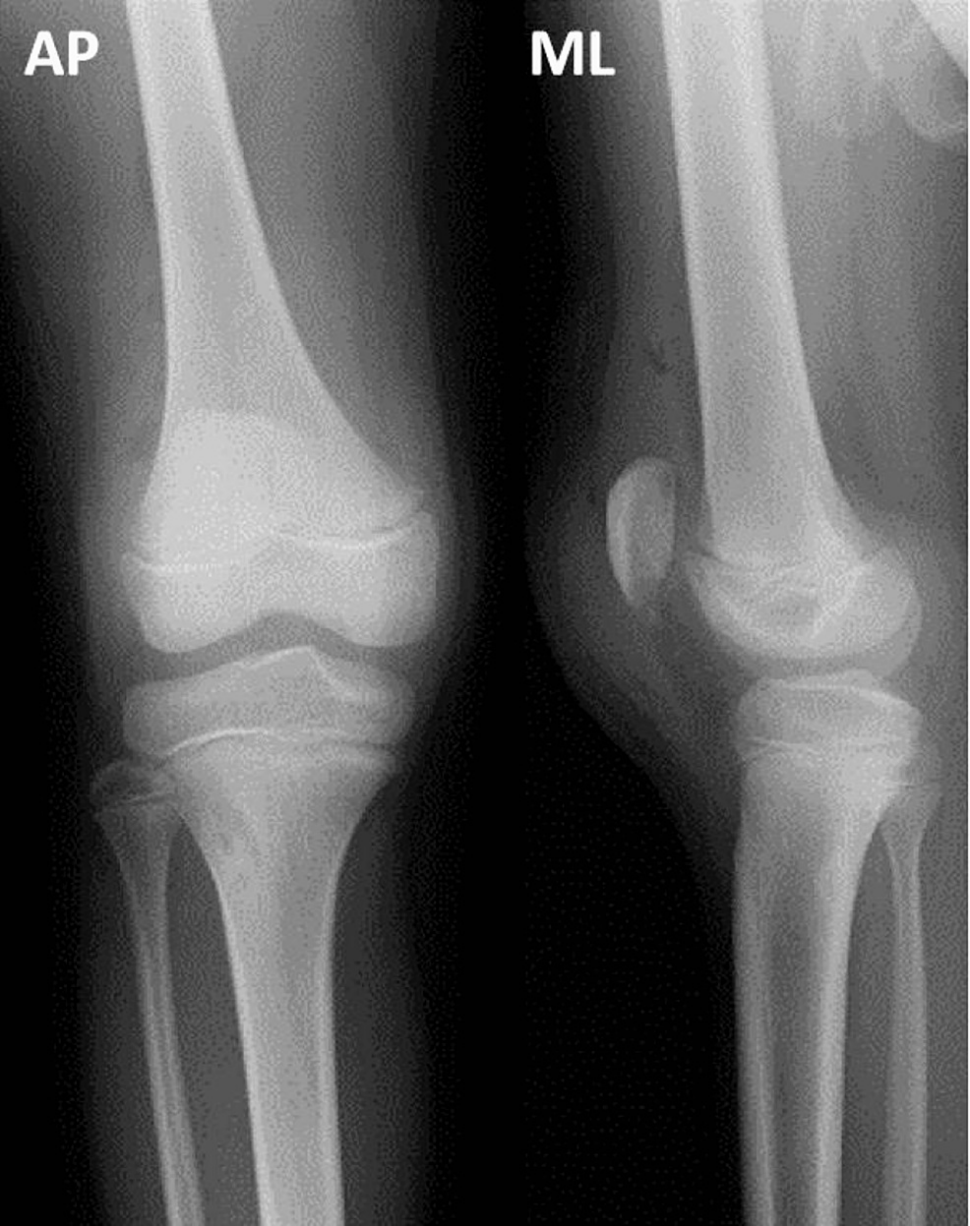
Plain radiographs obtained immediately after the operation. Good reduction and fixation were achieved.

We repositioned both the articular cartilage and the inferior pole of the patella with ligaments as much as possible and fixed them using the pull-out technique with no. 2 MaxBraid™ sutures (Figures 5-6).

A knee brace was applied with 10° flexion after the surgery to prevent deep knee flexion. Four weeks later, the knee brace was removed, and the intensity of physical therapy on the knee joint gradually increased. The patient was allowed full weight-bearing movement depending on his pain.

**Figure 7:**
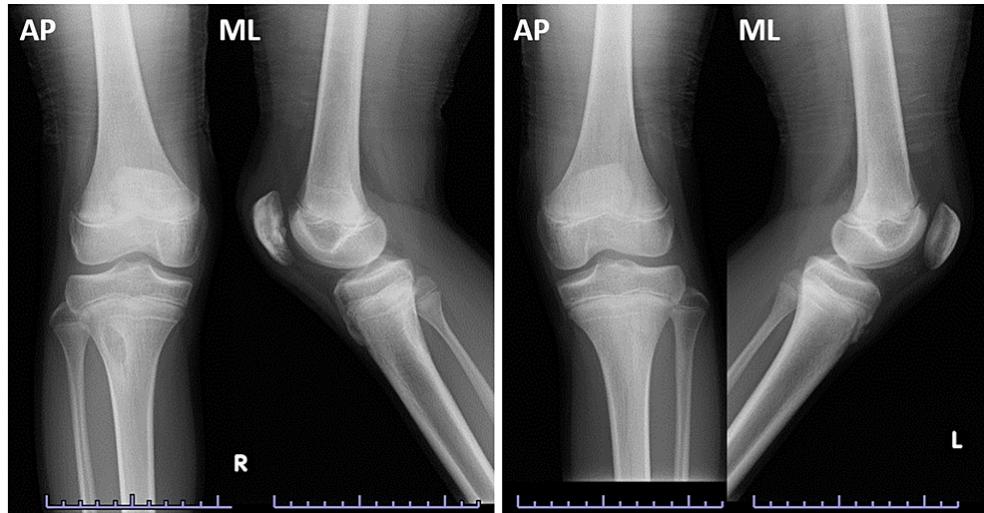
Plain radiographs at ten months postoperatively (left) and the contralateral side (right). Bone union was achieved. Patella height was almost same as the other side.

Compared to the patella height of the contralateral side, there was no difference at the final follow-up (Figure 7). Now, ten months after the surgery, the patient is able to exercise freely and move his knee joint fully again, without any extension lag.

At the final follow-up, the patient could exercise freely and move his knee joint fully, without any extension lag.

## Discussion

In terms of fractures among children, approximately 1% of all fractures occur in the patella, and 57% of these occur in the immature skeleton; therefore, patella fractures in children are relatively rare [2,3]. Children have skeletally immature patellae surrounded by a layer of articular cartilage and soft tissue, and ossification begins at the age of 3-6 years. Owing to this unique anatomical structure, children are prone to acute dislocation of the patella, which causes some fractures, particularly osteochondral, small peripheral, and sleeve-type fractures. In general, they usually occur in older children. The age of peak incidence for them is 12.7 years old, with a range of 8 to 16 years old. The high cartilage-to-bone ratio at the transformation zone makes it more susceptible to shear forces and eccentric loads, which can result in patellar sleeve fractures [4].

This trauma occurs due to a rapid contraction of the quadriceps muscle as an indirect force on a flexed knee, for example, when landing on foot. This causes a patella avulsion fracture with a lesion of the articular cartilage, periosteum, and patellar retinaculum [5].

This trauma is difficult to diagnose in an emergency room using only physical and radiographic examination, as it may be almost impossible to accurately evaluate the extensor mechanism of the knee owing to the severe pain and swelling caused by hemarthrosis. Moreover, most patellar sleeve fractures lack specific radiographic features, except for a high-riding patella, or patella alta, visualised on lateral plain radiographs. When a patellar sleeve fracture is suspected, other imaging modalities should be used to confirm its diagnosis. Ultrasound is one of the easiest and most affordable methods, but its success greatly depends on the operator’s skill [6].

According to recent studies, MRI is also a useful tool in its diagnosis [7,8]. In particular, MRI enables confirmation of the discontinuity between the patellar ligament and the patella as well as visualising the avulsed articular cartilage, which is not visible on plain radiographs. However, it is impossible to perform CT or MRI for all emergency room patients who present with knee pain of an unknown cause, owing to the high cost of these procedures. Nevertheless, a delayed or incorrect diagnosis can lead to a disturbance in the growth of the patella and a breakdown of the extensor mechanism of the knee. Thus, accurate and early diagnoses are essential to ensure appropriate treatment [9,10].

The treatment of patellar sleeve fractures depends on the degree of displacement. When the fracture displacement is less than 2 mm, conservative treatment with a cylindrical plaster of Paris cast can be selected. When the fracture displacement is more than 2 mm, surgical reconstruction of the knee’s extensor mechanism should be considered [11,12]. Owing to the small size of the fracture fragments in this type of trauma, selecting a method or material for internal fixation is often difficult. Various techniques for anatomical reduction and rigid fixation have been reported, including tension band wiring, transosseous sutures, intraosseous anchor sutures, and wire fixation as an augmentation, depending on the size of the osteochondral fragment [1,13,14]. If the fragment is too small to fix rigidly, transosseous or intraosseous anchor sutures should be considered [15].

Although the application of physical therapy has been controversial, various physical therapy techniques have been reported. Some authors demonstrate the need to achieve immobilization and prevent knee flexion for three to six weeks postoperatively; others claim that it is unnecessary to limit the range of movement or weight-bearing capacity [3,15,16].

Complications of this trauma include nonunion, degenerative changes, hypertrophic changes, transient ischaemic changes, the development of extensive ectopic bone within the knee, a limited range of movement in the knee joint, and patella alta [3,17].

In the present case, the patient was injured as he landed on the ground. The strong and rapid constriction force might have affected his quadriceps muscle in the flexed knee. Fortunately, we were able to reach an early and accurate diagnosis, as the emergency physician in our hospital acquired both CT and MRI scans before consulting with us about his condition.

Surgical treatment was the clear treatment choice owing to more than 2 mm of displacement at the fracture site. However, we further conducted a literature search for the best fixation method because the osteochondral fragment, in this case, was small and thin. Concerning several reports, we opted for the pull-out technique using transosseous No. 2 MaxBraid™ sutures, which were considered suitable for this method because of their strength and smoothness. We also used Krackow sutures [18] using a continuous locking loop technique commonly used in tendon repair. We did not use any wire because we considered the cheese-cutting phenomenon, which leads to loosening fixation, irritation for the skin, and further surgery for wire removal. We instead achieved a strong fixation and stability using the pull-out technique with a strong suture. While postoperative weight-bearing was permitted depending on the patient’s pain level, the knee joint was immobilized in a brace for four weeks to protect the knee from flexion.

## Conclusions

A patellar sleeve fracture is a rare trauma that occurs among children mainly because of the rapid contraction of the quadriceps in a flexed knee. Although this trauma can be easily missed on plain X-ray film due to the bony component being either very small or not present, MRI is one of the most helpful tools for diagnosis. Following a review of previous literature, early surgical intervention is considered essential for displaced cases. Various techniques for anatomical reduction and rigid fixation have been reported, including tension band wiring, transosseous sutures, intraosseous anchor sutures, and wire fixation as an augmentation, depending on the size of the osteochondral fragment. As demonstrated in our case, using the pull-out technique should be considered an effective option that can provide rigidity and safety. However, long-term assessments are required to identify any long-term postoperative complications such as quadriceps weakness, patella elongation or duplication, and long-term mobility issues.
